# Prevalence, prescribing and barriers to effective management of hypertension in older populations: a narrative review

**DOI:** 10.1186/s40545-015-0042-6

**Published:** 2015-10-14

**Authors:** Tariq M Alhawassi, Ines Krass, Lisa G Pont

**Affiliations:** Faculty of Pharmacy, University of Sydney, Sydney, Australia; College of Pharmacy, King Saud University, Riyadh, Saudi Arabia; Centre for Health Systems and Safety Research, Australian Insititue of Health Innovation, Macquarie University, North Ryde, Australia

**Keywords:** Hypertension, Older adults, Prevalence, Pharmacotherapy, Drug utilization

## Abstract

**Objectives:**

Hypertension is the leading modifiable cause of mortality worldwide. Unlike many conditions where limited evidence exists for management of older individuals, multiple large, robust trials have provided a solid evidence-base regarding the management of hypertension in older adults. Understanding the impact of age on how the prevalence of hypertension and the role of pharmacotherapy in managing hypertension among older persons is a critical element is the provision of optimal health care for older populations. The aim of this study was to explore how the prevalence of hypertension changes with age, the evidence regarding pharmacological management in older adults and to identify known barriers to the optimal management of hypertension in older patients.

**Methods:**

A review of English language studies published prior to 2013 in Medline, Embase and Google scholar was conducted. Key search terms included hypertension, pharmacotherapy, and aged.

**Results:**

The prevalence of hypertension was shown to increase with age, however there is good evidence for the use of a number of pharmacological agents to control blood pressure in older populations. System, physician and patient related barriers to optimal blood pressure control were identified.

**Conclusions:**

Despite good evidence for pharmacological management of hypertension among olderpopulations, under treatment of hypertension is an issue. Concerns regarding adverse effects appearcentral to under treatment of hypertension among older populations.

## Introduction

Population aging has considerable implications for health care systems internationally, especially from the perspective of pharmaceutical practice and policy. “Older adults” are commonly defined in the medical literature as individuals aged 65 years or over [1]. Internationally, the proportion of the population defined as older adults is increasing, primarily as a result of the increase in life expectancy [2]. By 2050 it is anticipated that older population will account for 21 % of the total population in most developed countries [3], while in some countries, such as Japan, older adults already represent one-fifth of the total population [4, 5]. A similar increase applies to the very old adults (age ≥80 years), one of the fastest growing segments of older adults population and one that is expected to triple by 2050 [6].

Hypertension is the leading modifiable cause of mortality worldwide [7]. Unlike many conditions where limited evidence exists for management of those aged over 65 years due to the exclusion of older populations from clinical trials [8, 9], the findings of multiple large, robust trials have provided a solid evidence-base regarding the management of hypertension in older adults [7]. Adverse outcomes associated with poor blood pressure (BP) control in older persons have been well documented. A Cochrane review of 12 clinical trials showed that the management of hypertension in people aged 60 years and over was associated with a reduction in mortality (Relative Risk (RR)) = 0.9, 95 % confidence interval ((CI) 0.84–0.97) [10]. The same review reported pharmacological management of hypertension in older adults was associated with significant reductions in both cardiovascular (RR = 0.77, 95 % CI 0.68–0.86) and cerebrovascular mortality (RR = 0.66, 95 % CI 0.53–0.82) [10].

Given the expected increase in the older adult population and the wealth of evidence regarding the management of hypertension in older persons, understanding how the prevalence of hypertension changes with age as well as how physicians currently manage older patients with increased BP is important. Furthermore, insight into current barriers to the provision of optimal management is essential if clinicians are to meet the health needs of the growing older populations. The aim of this study was to provide an overview of the prevalence of hypertension and its pharmacological management in older adults. A secondary aim was to explore known barriers to the optimal management of hypertension in older patients.

## Methods

Narrative review methodology as described by Green *et al.* [11] was used to conduct a non-systematic narrative review of the literature regarding pharmacotherapy for the management of hypertension in older populations.

### Data sources and study selection

An electronic search of the electronic databases EMBASE, MEDLINE for studies published prior to 2013 regarding the use of pharmacotherapy for the pharmacological management of hypertension among older populations was conducted. In addition, Google scholar was searched to identify any non-indexed relevant publications. Search terms used included: hypertension, pharmacotherapy, and aged. Additional search terms used included: drug therapy, elderly, older patients, and geriatric. Search terms were mapped to MESH headings in Medline and EMTREE headings in Embase. Studies which focused specifically on pharmacological management of hypertension in older persons were included in the review. Exclusion criteria were non-English language studies, studies in non-human populations as well as studies that did not specifically focus on populations aged 65 years and older or on pharmacological management of hypertension.

### Data synthesis

A narrative synthesis of the prevalence of hypertension in older populations, the evidence regarding the use of different pharmacological agents and identification of barrier to optimal pharmacological management of hypertension among older persons was conducted.

## Review

### Prevalence of hypertension in older populations

**Fig. 1:**
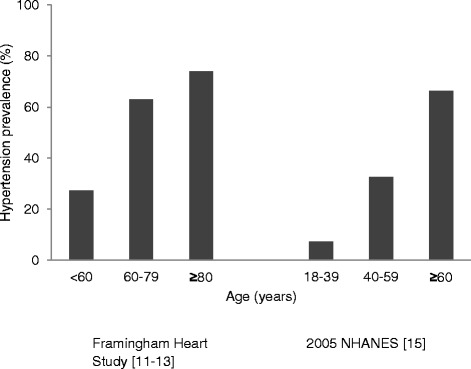
Increasing prevalence of hypertension with age

Data from the Framingham study in 1978 and 2002 [12–14] and the 2005 US National Health and Nutrition Survey (NHANES) [15] have shown clear increases in the prevalence of hypertension with age (Fig. 1). The original Framingham study followed 5209 respondents from 1948 until 2005, exploring the development of cardiovascular disease and identification of associated risk factors over time [2, 3]. The NHANES surveys are annual cross sectional surveys which combine interview and physical examination, to assess health status across representative samples of the American population.[15] The NHANES data demonstrated that increases in the prevalence of hypertension prevalence begin in adulthood, with the prevalence doubling between the ages of 20–40 years, and then with a further 100 % increase occurring between 40 and 60 years. The Framingham study showed that this pattern continues as people age, with the prevalence of hypertension increasing from 27.3 % in those aged ≥ 60 years to 74.0 % in those aged over 80 years.

Gender differences in the prevalence of hypertension have been noted in both younger and older populations. A number of studies have shown found that although women have a lower prevalence of hypertension than men at a younger ages, this pattern changes after the age of 60 years when the pattern reverses with the prevalence in women exceeding that in men.[13, 16–21],

Differences have been reported in terms of aging with respect to increases in systolic blood pressure (SBP) compared with diastolic blood pressure (DBP). Both systolic and diastolic pressure have been reported to increase with age. [15] This increase continues until the ninth decade however it is believed that DBP may plateau or even decrease around the age of 60 years. [15] These differences may account for the increase in isolated systolic hypertension that is associated with aging. Isolated systolic hypertension refers to elevated SBP with a normal DBP. [22] .

Pathophysiological changes including increased peripheral vascular resistance due to arterial stiffening, which occur with age, are believed to be one factor associated with the increase in hypertension associated with aging. [15, 23]. This increase in arterial stiffness with ageing is believed to alter the normal hemodynamic pattern of blood pressure (BP) causing an increased pulse wave velocity, an index of arterial stiffness, and widening pulse pressure therefore accounting for the observed age–related decline in DBP and increase in SBP. [17, 23–25]. Other factors such as the changes in renin and aldosterone levels, decreases in renal salt excretion, age-related declines in renal function and associated changes in the autonomic nervous system and endothelial function are also considered contributory factors.[16, 26–28]. Life style related factors might also contribute to the development of hypertension. Factors such as stress, excessive alcohol intake, sedentary life style, high sodium intake and obesity are risk factors for hypertension [16, 29–31]. Moreover a synergistic effect on the risk of hypertension has been observed when multiple factors exist synchronously [16, 29–31].

### Pharmacological management of hypertension in the elderly

While life-style interventions are generally the first-line strategy for the management of all persons with hypertension, the majority of hypertensive patients will require pharmacological intervention to adequately control their blood pressure. [32]. A number of large well-conducted clinical trials exploring pharmacological management of hypertension in the elderly have been conducted and there is good evidence for the use of a variety of different antihypertensive agents in the management of hypertension in the elderly. [10]

#### Thiazide and thiazide –like diuretics

Thiazides diuretics are one of the oldest drug classes used in the treatment of hypertension. [33] Evidence of effectiveness in lowering BP and preventing the cardio and cerebrovascular adverse outcomes associated with hypertension in the elderly has been supported by several clinical trials, including the Hypertension in the very elderly (HyVET) [34], the Swedish Trial in Old Patients with Hypertension (STOP)[35, 36] and the Antihypertensive and Lipid-Lowering Treatment to prevent Heart Attack Trial (ALLHAT) [37] studies While not all these studies focused specifically on the elderly, the mean participant age was over 65 years for all, and their recommendations were especially relevant for the younger elderly population.

Use of thiazide and thiazide like diuretics for the management of hypertension in older persons has declined over the past decade. [19, 20, 38–40]. There are a number of possible explanations for this change in the pattern of thiazide prescribing including increased use of other diuretics, particularly in elderly with complicated hypertension [41, 42], the advent of other antihypertensive medications such as Calcium Channel Blockers (CCBs) or agents acting on the Renin Angiotensin System (RAS), as well as greater caution by prescribers due to increased risk of adverse drug reactions in elderly [43, 44]. Yet, despite this general decline in thiazide use among older persons with hypertension, their use remains high [15, 45, 46], and they are the most commonly used agents in combination therapy in the management of hypertension. [19, 20, 38, 39, 45, 47].

#### Agents acting on the Renin-Angiotensin System (RAS)

There are three main three antihypertensive classes that act on RAS. These are the angiotensin receptor blockers (ARBs), the Angiotensin concerting enzyme inhibitors (ACEI) and the direct renin inhibitors. The use of both ARBs [15, 38, 45] and ACEIs [15, 19, 20, 38, 40, 45, 47–49] in the elderly is generally high, and has increased over years surpassing other antihypertensive classes such as CCBs. The increase in use of these agents has been supported by clinical trials such as the Second Australian National Blood Pressure (ANBP2) [50] which demonstrated that ACEI were superior to thiazide diuretics in terms of cardiovascular outcomes, however there was no difference between the regimens in terms of all cause mortality. In addition to increased use as monotherapy, ACEI and ARB use in combination with other antihypertensive medications has also increased over recent years [19, 20, 38, 39, 45, 47]. In contrast to the common use of ACEIs and ARBs, use of aliskiren, a direct renin inhibitor that has been approved for use since 2007 in elderly hypertension patients is minimal[15, 47]. The slow uptake of aliskiren for use among the elderly may be hindered by its limited efficacy and relatively poor safety profile.[51, 52]

#### Calcium channel blockers (CCBs)

Since the introduction of CCBs, the prescribing pattern of this antihypertensive medication class in the elderly has increased both as monotherapy and combination therapy. [15, 20, 39, 48, 49] Despite publication of the Systolic Hypertension in the Europe Trial (SYST-EUR) in 1997 which showed that treating 1000 patients for 5 years with a CCB regimen prevents 29 strokes or 53 MIs [53], a decline in the use of CCBs has generally been noticed in elderly and very elderly patients since the mid 1990s has been observed. [19, 38, 45] This decline in use may be secondary to proposed safety concerns including increased risk of cancer, myocardial infarction and gastrointestinal heamorrhage with long term use [54–56].

#### Beta blockers (BBs)

Beta blockers were among the most commonly prescribed agents since their introduction as an option for the treatment of hypertension [40, 47, 48]. However, use in the elderly has decreased [19, 20, 38, 39, 43]. following publication of a meta-analyses raising questions about their efficacy and highlighting safety concerns with an increased risk of stroke reported with BB use as monotherapy [57]. The current low rate of use was highlighted in a recently published observational study. [45]

#### Alpha blockers

While several studies have shown a slight increase in the use of alpha blockers in the management of hypertension in the elderly and very elderly patients [15, 40], overall their use has declined [20, 45, 47, 48]. This decline in use may be due to the poor adverse effect profile of the alpha blockers in the elderly as well as to a lack of evidence in preventing cardiovascular related complications when compared with other antihypertensive agents.

### Barriers to the optimal management of hypertension in the elderly

Despite the wealth of evidence regarding the benefits of managing hypertension in older and very old populations, a substantial proportion of older persons have suboptimal blood pressure control. [58]. A number of barriers to optimal blood pressure control in the elderly have been identified and these barriers can generally be considered as system-, physician- or patient-related barriers.

System–related barriers affecting blood pressure control in the elderly include the variability in treatment recommendations for this population. [59, 60]. While a number of clinical trials have been conducted in the elderly, the extent to which this evidence has been incorporated into treatment guidelines and translated into practice remains unknown.

Physician-related barriers include differences in physician attitudes towards the risks and benefits of managing hypertension in older persons as well as differences in interpretation of the evidence. [61–64]. Inconsistency between treatment guidelines regarding management of hypertension in the elderly may make it difficult for clinicians to incorporate the evidence-based recommendations in their daily clinical practice. [61, 62, 65]. The lack of consistency in guidelines may also influence prescribing decisions in the treatment of complex patients, such as those with multiple co-morbidities. [61, 63, 66, 67]

Patient awareness of their condition may also influence medication use and BP control. Patients with a higher awareness of the need for regular blood pressure monitoring are more likely to have controlled blood pressure, [63, 66–70] while those with poorer adherence to prescribed medication are less likely to have controlled BP. [18, 71–74]

Adverse drug reactions may influence both the clinician’s decision to prescribe a medication[75, 76], as well as the patient’s decision to continue using a medication [66], and more work is needed to better understand the role adverse drug reactions play in the use of antihypertensive medications in the elderly.

Unlike a systeamtic review where the aim is to capture all revelant literature, as a non-systematic narrative review, the aim of this study was to provide an wide overview of the prevalence of hypertension and its pharmacological management in older adults. To cover a wide range of published literature, searches were conducted in both Medline and Embase electronic databases. In addition, a general search of Google scholar was also conducted to capture relevant published work not indexed in either Medline or Embase. However inclusion of other databases may have widened the range of literature included in the analysis and is one limitation of this work.

## Conclusion

The prevalence of hypertension increases significantly with age. While approximately 30 % of the adult population aged less than 65 years has hypertension, by the age of 80 over 70 % of the population has or is being treated for elevated blood pressure. The estimated direct and indirect economic impacts and medical expenditure of hypertension is high and, given predicted increases in the elderly population, likely to increase. Despite the availability of effective antihypertensive medications and good evidence for blood pressure reduction in older populations, current management, management appears suboptimal. Barriers to optimal blood pressure control in older populations are often multifactorial highlighting the clinical complexity of this patient population. System, prescriber and patient related barriers to the management of hypertension exist and is interplay between these such as the lack of clinical guidance regarding management of hypertension in complex older patients with multiple comorbidities contribute to suboptimal blood pressure control in older populations.

## Abbreviations

RR: Relative risk
CI: Confidence interval
SBP: Systolic blood Pressure
DBP: Diastolic blood pressure
BP: Blood pressure
RAS: Renin angiotensin system
ARB: Angiotensin receptor blocker
ACEI: Angiotensin concerting enzyme inhibitor
CCB: Calcium channel blocker
BB: Beta blocker
